# The Risk of Paradoxical Embolism (RoPE) Study: Developing risk models for application to ongoing randomized trials of percutaneous patent foramen ovale closure for cryptogenic stroke

**DOI:** 10.1186/1745-6215-12-185

**Published:** 2011-07-27

**Authors:** David M Kent, David E Thaler

**Affiliations:** 1 Institute for Clinical Research and Health Policy Studies, Tufts Medical Center/Tufts University School of Medicine, Boston, MA, 02111, USA; 2 Department of Neurology, Tufts Medical Center/Tufts University School of Medicine, Boston, MA, 02111, USA

## Abstract

**Background:**

Despite the diffusion into practice of percutaneous closure of a patent foramen ovale (PFO) in patients with cryptogenic stroke (CS), the benefits have not been demonstrated, and remain unclear. For any individual presenting with a PFO in the setting of CS, it is not clear whether the PFO is pathogenically-related to the index event or an incidental finding. Further, the overall rate of stroke recurrence is low in patients with CS and PFO. How patient-specific factors affect the likelihood that a discovered PFO is related to an index stroke or affect the risk of recurrence is not well understood. These probabilities are likely to be important determinants of the benefits of PFO closure in CS.

**Design/Methods:**

The goal of the Risk of Paradoxical Embolism (RoPE) Study is to develop and test a set of predictive models that can identify those patients most likely to benefit from preventive treatments for PFO-related stroke recurrence, such as PFO closure. To do this, we will construct a database of patients with CS, both with and without PFO, by combining existing cohort studies. We will use this pooled database to identify patient characteristics associated with the presence (versus the absence) of a PFO, and to use this "PFO propensity" to estimate the patient-specific probability that a PFO was pathogenically related to the index stroke (Model #1). We will also develop, among patients with both a CS and a PFO, a predictive model to estimate patient-specific stroke recurrence risk based on clinical, radiographic and echocardiographic characteristics. (Model #2). We will then combine Models #1 and #2 into a composite index that can rank patients with CS and PFO by their conditional probability that their PFO was pathogenically related to the index stroke *and *the risk of stroke recurrence. Finally, we will apply this composite index to completed clinical trials (currently on-going) testing endovascular PFO closure against medical therapy, to stratify patients from low-expected-benefit to high-expected-benefit.

## Background

Approximately 40% of all strokes are classified as cryptogenic, meaning that the cause is unknown despite an extensive work up [1]. While patent foramen ovale (PFO) is a common and generally benign condition found on autopsy in about 25% of the population [2-4], approximately 40% to 50% of patients younger than age 55 with cryptogenic stroke (CS) have PFO on transesophageal echocardiography (TEE) [4-7]. Furthermore, a PFO is found more frequently in patients with CS than in patients with a known cause of stroke, even in the elderly patient[7-9], These association suggest that PFO has an etiological role in CS, presumably via paradoxical emboli (PE) (i.e. venous emboli that gain access to the arterial circulation through a PFO) [7]. Thus, many physicians will recommend PFO closure in patients, especially younger patients, who have had CS.

However, the benefit of PFO closure in patients with CS has not been demonstrated, and remains unclear. First, for a patient presenting with a PFO and a CS, it is not clear for that individual whether the PFO is causally related to the index event or whether it is an incidental finding, since PFOs are so common in the general population. Second, the overall rate of stroke recurrence is relatively low in patients with CS and PFO, ranging in studies from 0% to 12% per year, with an average annualized risk across studies of approximately 2% [4,5,10]. Thus, even rare but serious procedure- and device-related complications may be sufficient to nullify the benefit of percutaneous closure, especially if many who receive the device have index strokes unrelated to their PFO. A method is needed to identify patients with CS and pathogenic PFOs, especially those with a high risk of recurrence from patients with CS and incidental PFOs.

**Figure 1:**
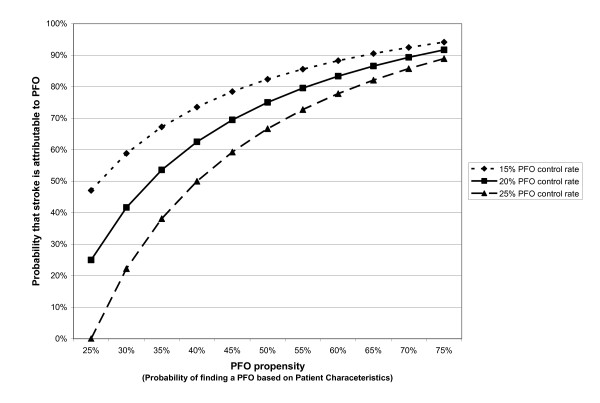
**PFO Propensity and the Probability that a Stroke is PFO-attributable**. We define PFO propensity as the probability of finding a PFO in a patient, based on patient-specific characteristics (such as age and the presence or absence of hypertension, diabetes and hypercholesterolemia). Through Bayes' theorem, it is directly (though non-linearly) related to the probability that a cryptogenic stroke is PFO-attributable (in patients with both cryptogenic stroke and PFO).

The theoretical rationale for the RoPE study has been described in prior work [7,11]. Briefly, the RoPE Study builds on prior work demonstrating the potential importance of risk modeling in the interpretation of clinical trials, where overall results may not reflect the benefits to individual patients [12-17]. In the setting of CS specifically, we have shown that the probability that an individual will have a PFO can be predicted (i.e. prior to TEE) based on the presence or absence of conventional stroke risk factors (e.g. age, presence of hypertension, diabetes, hypercholesterolemia, etc.) and other characteristics. We call the patient-specific probability of finding a PFO in the setting of CS the "PFO propensity". Using simple assumptions and Bayes' theorem, PFO propensity can be shown to be directly but not linearly related to the patient-specific PFO-attributable fraction (i.e. the probability that the PFO is pathogenic rather than incidental)-- as shown in the Figure 1. Thus, although it is not possible in the individual patient to determine with certainty whether a discovered PFO is incidental or pathogenic, it is nonetheless possible to construct mathematical models that can stratify patients by their likelihood that their stroke is PFO-related.

Additionally, while prior studies have had limited statistical power to develop predictive models that can predict stroke recurrence given the low frequency of this outcome, the RoPE Study aims to overcome this barrier by combining multiple large multicentered studies to yield a pooled database with a sufficient number of cerebrovascular outcome events to model recurrence risk.

**Figure 2:**
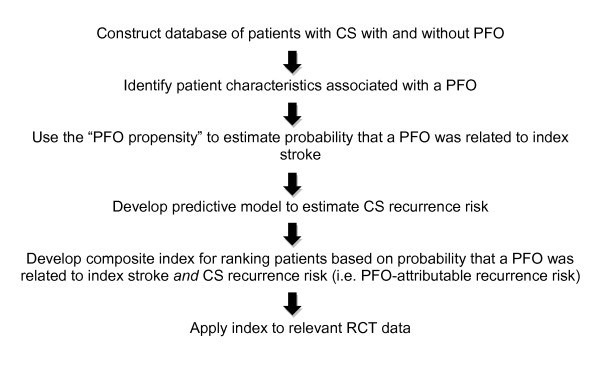
**Summary of RoPE Study**. Schematic summary of the Risk of Paradoxical Embolism (RoPE) Study.

The aim of the RoPE Study is to develop mathematical models that can be used to stratify patients by the joint probability that an index stroke is "PFO-related" and that the stroke will recur and to use these models to stratify patients in on-going clinical trials testing percutaneous closure, with the goal of informing patient-selection for PFO closure in clinical practice. The steps by which this will be accomplished are summarized in Figure 2, as well as in the specific aims below.

## Design/Methods

The specific aims of the RoPE Study are:

1. To construct from existing cohort studies of patients with CS studied with TEE, both with and without PFO, the largest extant CS database, sufficiently robust to support predictive risk modeling.

2. To identify patient characteristics associated with the presence (versus the absence) of a PFO, and to use this "PFO propensity" to estimate the patient-specific probability that a PFO was pathogenically related to the index stroke. (Model #1)

3. To develop a predictive model to estimate patient-specific stroke recurrence risk based on clinical, radiographic and echocardiographic characteristics in patients with PFO and CS. (Model #2)

4. To develop a composite index based on Models #1 & #2 that can rank patients with CS and PFO by their conditional probability that the PFO was pathogenically related to the index stroke (Model #1) *and *the risk of stroke recurrence (Model #2).

5. To apply this composite index to patients in completed clinical trials (currently on-going) testing endovascular PFO closure against medical therapy, to stratify them from low-expected-benefit to high-expected-benefit.

### Aim 1: To construct the largest extant CS database from existing cohort studies of patients with CS studied with TEE for the presence/absence of PFO

Methods for pooling multiple independent studies have been previously described [18,19]. Steps include: 1) selection of participating clinical trials and databases; 2) development of a collaborative investigative team including clinicians, statisticians and programmers; 3) preliminary determination of the availability and characteristics of data in each of the component databases; 4) complete specification of the dependent (outcome) variable used in each study and in the pooled analyses; 5) determination and specification of the independent (explanatory) variables; 6) specification of the inclusion/exclusion criteria for the studies; 7) verification of variable definitions; 8) acquisition of new data if required; 9) checking for consistency of effects across study databases.

Based on our own literature search, a published literature review [4], and then subsequently on a published meta-analysis [10], we contacted all investigators with relevant data examining the rate of recurrent stroke in patients CS and PFO. This also yielded data examining the presence/absence of PFO in patients with CS. We obtained agreements to include 13 databases with over 2500 subjects, as shown in Table 1. This provides the basis of our power calculations for subsequent aims.

**Table 1:** RoPE Database

Database	# of subjects	# w/PFO	# w/o PFO	RoPE Investigator
APRIS [6]	83	17	66	Marco DiTullio
Bern (published) [30]	159	159	-	Krassen Nedeltchev
Bern (unpublished)	249	249	-	Heinrich Mattle
CODICIA [31]	486	266	240	Joaquin Serena
PFO-ASA Study [5]	581	267	314	Jean-Louis Mas
German Study [24]	1126	389	737	Christian Weimar
Lausanne	172	93	79	Patrik Michel
NOMASS [32]	60	23	37	Mitchell Elkind
PICSS [33]	250	98	152	Shunichi Homma
Sapienza (unpublished)	343	133	210	Emanuele Di Angelantonio
				Federica Papetti
Toronto [34]	114	114	-	Cheryl Jaigobin
Tufts	140	140	-	David Thaler
Total Estimates*	2546	1484	1062	

Precise determination of inclusion/exclusion criteria, specification of the independent variables and specification of the dependent variable for each of the models are dependent on careful examination of each of the component databases for details of variable definition and missingness. s Consensus on the variables will be reached among RoPE Investigators for the final models. Provisional variable descriptions are provided below.

The RoPE Study will include patients with CS who have been examined for the presence of a PFO with either TEE or Transcranial Doppler (TCD) and the injection of bubbles. The preliminary definition of CS for the RoPE Study conforms to the Trial of ORG 10172 in Acute Stroke Treatment (TOAST) classification,[20] and requires a "complete work-up" (defined below) for the cause of the stroke. Cardioembolic stroke in this classification was considered "probable" if there was a high-risk source of embolism and "possible" for medium risk sources. This latter category includes PFO and atrial septal aneurysms (ASA). For the purposes of the RoPE Study, subjects with medium risk sources will be considered cryptogenic. The definition of stroke for the RoPE Study is *"a sudden onset neurological deficit in a vascular territory presumed to be due to focal ischemia*." If the deficit lasts for less than 24 hours, it must be accompanied by acute magnetic resonance imaging (MRI) or computer tomography (CT) changes in appropriate locations. If the deficit is greater than 24 hours, MRI or CT changes are not required. *A "complete work-up" *must include: 1) MRI *or *CT; 2) Vascular imaging - catheter angiography or angiography by MRI or CT; 3) Cardiac rhythm study: at least one electrocardiography (ECG), or documented Holter or telemetry.

In addition to excluding patients with stroke of known cause, patients included in the RoPE study must be worked up for the presence of a PFO with a TEE using intravenous injection of bubbles. Patients can be included in model #1 without a TEE, if they have had both transthoracic echocardiography [to rule out other potential cardioembolic sources] and TCD with bubbles (to establish the presence/absence of right-to-left shunting). Only patients with a TEE will be included in Model #2, since this model will require assessment of the presence/absence of ASA and PFO grading.

Table 2 shows provisional independent (predictor) variables that we anticipate will be important in both the prediction of the presence/absence of PFO, and also the risk of the outcome (recurrent ischemic stroke, as defined above). Of note, we anticipate that harmonization of databases may require the acquisition of new primary data, potentially from the following sources: 1) medical record review 2) rereading of TEE tapes; 3) rereading of neuroimages; 4) obtaining follow-up for recurrent events. In general, the reasons for needing additional primary data from a particular database may include: 1) missing data on a portion of patients; 2) differing variable definitions between databases; and 3) new data (variables) required not previously collected in a given database. Medical record review would be possible for clinical registries and TEE tapes and neuroimages are potentially available for most of the component databases. Based on the characteristics of any observed missing data we will investigate the use of imputation and replacement algorithms, or whether a component database needs to be excluded--although statistical methods to preserve data and power will be fully employed (such as imputation for independent variables). Because missingness of the dependent (outcome) variable poses more potential problems due to informative censoring, we intend to exclude any datasets for which less than 90% of patients have follow-up at one year. Re-adjudication of all stroke outcomes is planned as part of the RoPE study. Methods ensuring database quality during database construction will be complemented by steps taken during the predictive modeling process to check for consistency of effects across databases.

**Table 2:** Initial RoPE Variables of Interest

Variable Type	Variable
**Clinical**	Age
	Gender
	Race
	History of the following (prior to index stroke)
	*Migraines*
	*Hypertension*
	*Diabetes*
	*Prior cerebral ischemia*
	*Coronary artery disease*
	*Obesity*
	*Hypercholesterolemia*
	*Smoking status*
	*Antithrombotic medications*
	*Deep vein thrombosis*
	*Pulmonary Embolism*
	*Hypercoagulable states*
	Antithrombotic medications after index event
	Outcome: National Institutes of Health Stroke Severity Score

**Echocardiographic**	Hypermobility of interatrial septum ("ASA")
	Interatrial shunting at rest (e.g. not during Valsalva maneuvers)
	Volume of interatrial shunt (e.g. max. bubbles in left atrium)
	Anatomical PFO size
	Spontaneous Doppler flow seen on color

**Radiological**	MRI/CT findings of cerebral infarct: yes/no (at index stroke)
	Number of prior cerebral infarcts
	Anatomical location of index and prior infarct(s)

### Aim 2: To identify patient characteristics associated with the presence (versus the absence) of a PFO, and to use this "PFO propensity" to estimate the patient-specific probability that a PFO was pathogenically related to the index stroke. (Model #1)

Since PFOs discovered in patients with CS may be pathogenically related to the stroke or not, in Aim 2, we will use logistic regression models to identify patient characteristics that are associated with the presence of a PFO among patients with CS ("PFO propensity"). In previous work [7,11,21], we have shown that this "PFO propensity" can be related to the probability that a PFO is pathogenic (i.e. non-incidental) using Bayes' theorem and 2 simple assumptions: 1) if not for those strokes that are PFO-attributable, the probability of a PFO in a CS patient would be the same as in the general population (controls); and 2) the rate of PFO-attributable strokes in PFO-negative CS patients is near-zero. Under these conditions:

Since the patient-specific probability of PFO in CS is given by the PFO propensity (i.e. the prevalence of PFO in CS patients otherwise similar to the patient), the only additional term needed to estimate the probability that a PFO is incidental for a particular patient is the general rate of PFO in the population (PFO probability in controls).

To develop a predictive model for PFO propensity, we will explore crude associations (overall and within each database) between the presence of a PFO and clinical variables including the subject's age, gender, race, hypertension, diabetes, hyperlipidemia, history of prior episodes of cerebral ischemia, obesity, antithrombotic medications, concurrent deep venous thrombosis, and neuroradiologic variables (i.e. prior stroke on MRI or CT, small [< 1.5 cm] versus large infarct, location of infarct). Multivariable associations between predictors and the presence of a PFO will be examined using logistic regression models. Selection of candidate variables for these models will be based on clinical rationale and the published literature. Variables with statistically marginal associations in the preliminary analyses (p > 0.15) will be removed. Non-linear associations between independent risk variables and the outcome will be investigated using generalized additive spline models[22]. As indicated, appropriate parametric logistic regression models will be developed to capture these non-linear relationships.

Statistical significance of individual variables in the final model will be assessed with the Score statistic. Based on clinical reasoning, a limited number of interaction terms between risk factors may also be tested for inclusion in the model. The models will be analyzed by conventional criteria, such as goodness-of-fit tests, and receiver operator characteristics (ROC) curve areas [23]. "Calibration" of predicted probabilities of outcome throughout the range of their predictions will be evaluated with plots. The final multivariable regression models will be run separately on each of the component datasets to assess the consistency of the results and model performance. Based on these results, interaction terms between indicator variables representing study and model predictors of PFO will be used to investigate significant variation in associations across study datasets. Predictors found to be inconsistent across studies may be removed from the model. We will reanalyze the data using a generalized linear mixed model that includes a random effect term for study to get final parameter estimates with their corresponding standard errors.

Adequacy of the Data for Aim 2: Based on a preliminary review of the included studies (see Table 1), we will have potentially 3,101 subjects available for the models predicting PFO (1,332 with and 1,769 without PFOs), drawn only from those databases enrolling all cryptogenic stroke patients with and without PFO. This sample size will provide 80% power to detect an odds ratio of 1.23 for a balanced risk factor. For a risk factor with a prevalence of 20%, this sample size will still provide 80% power to detect an odds ratio of 1.29. The study will still provide sufficient power to detect clinically relevant associations even in the presence of significant data being unavailable for modeling. For example, if we totally eliminate data from the largest RoPE component study (the German database [24]), we will have 1,975 included subjects (928 with and 1047 without PFOs.) This sample will provide 80% power to detect odds ratios of 1.29 and 1.38 for risk factors with a prevalence of 0.50 and 0.20, respectively. Since outcome data is not needed for this analysis, loss of this amount of data is unlikely and the database would anyway still have ample power, permitting full exploration of multiple variables.

### Aim 3. To develop a predictive model to estimate patient-specific recurrence risk based on clinical, radiographic and echocardiographic characteristics in patients with PFO and CS. (Model #2)

For Aim 3 we will use Cox proportional hazard models to develop a predictive model to estimate patient-specific recurrence risk. Because patients with CS with higher PFO-related recurrence risk generally have a lower prevalence of conventional stroke risk factors[25], failure to adjust for general (non-PFO-related) recurrent risk can lead to paradoxical results. This has been a major challenge for prior studies, since the low rate of outcomes limits the statistical power to control for conventional stroke risk factors and model PFO-related risk. While in the RoPE study we will have many-fold the number of outcomes compared to any prior study, statistical power for this analysis remains an issue.

To address this, depending in part on the number of outcomes, we will consider a two-step approach, that takes advantage of the outcomes in the component of the RoPE database of CS patients *without *PFO. First, we will construct a model for recurrence risk on the component of the study of CS patients *without *PFO. Next, we will construct a model for recurrence risk on the component of the study of CS patients *with *PFO, using the derived risk variable from the first step to control for the confounding background risk of stroke from PFO-unrelated causes. This will permit background stroke risk to be controlled for in a statistically efficient fashion. For step two, using the study subjects with known PFO, we will pay particular attention to echocardiographic information (e.g. hypermobility of the interatrial septum, interatrial shunting at rest, severity of interatrial shunt, anatomical PFO size, presence/absence of spontaneous flow on color Doppler)--to identify features of "high-risk" PFOs.

Preliminary analyses for both phases of this two step process (i.e. modeling recurrence risk in patients without PFO and modeling recurrence risk in patients with PFO) will investigate associations between established risk factors and recurrent strokes. Kaplan-Meier survival curves and logrank tests will be used to assess statistical significance. For each phase, a multivariable prediction model for recurrent stroke will be developed using a Cox proportional hazard model, using an approach otherwise similar to that described for Aim 2. Predictive discrimination will be assessed using the overall C index [26]. Model calibration is assessed by comparing predicted survival probabilities against Kaplan-Meier (observed) probabilities across equal-sized risk quantiles (based on predicted recurrence probabilities). Differences between predicted and observed probabilities will be compared using a modified Hosmer-Lemeshow χ^2 ^statistic [27]. The Cox models be re-run including the study as a stratification factor to control for potential between-study differences in baseline survival functions when estimating hazard ratios of potential risk factors.

Adequacy of the data for Aim 3: Power was calculated on extreme optimistic and extreme pessimistic calculations. To obtain outcome rates for our sample we used databases with patients who had PFOs. We optimistically estimate available follow-up data for up to 1,752 patients with PFO; 1,332 from databases with both PFO and non-PFO patients and an additional 420 from databases that only have data on patients with PFOs. Based on the average outcome rates on the observed data, the estimated stroke rate for these patients is 6%. The sample size of 1,752 will provide 80% power to detect a clinically relevant hazard ratio of 1.77 for a risk factor with a prevalence of 50% and an overall stroke rate of 6%. Most pessimistically, we excluded 100% of our largest study (the German study [24]) and estimated follow-up will only be available for 70% of the remaining subjects which would result in a total sample size of 944. With this extremely conservative estimate of the sample size the study will have 80% power to detect a hazard ratio of 2.22 assuming an overall stroke rate of 6% and a risk factor prevalence of 50%.

While having a sample size toward this lower estimate would still be adequate to detect these moderate to large effects, it would require us to be parsimonious in testing variables to avoid over-fitting (only about 60 stroke outcomes in PFO patients). After final database construction, we will consider whether to model the risk of the composite outcome stroke and transient ischemic attack (TIA). This has an outcome rate of 11.2% on the sample of our database with completed follow-up, virtually doubling the number of outcomes.

### Aim 4. To develop a composite index that can rank patients with CS and PFO by their conditional probability that the PFO was pathogenically-related to the index stroke (Model #1) *and *the risk of stroke recurrence (Model #2)

For Aim 4, we will combine our probability models in order to stratify patients by their risk of stroke recurrence conditioned on the probability that the PFO is pathogenically-related (i.e. not incidental) to the index stroke. Thus, the aim of this step is to combine models to estimate the PFO-attributable recurrence risk. This step prepares us for Aim 5: stratification of the clinical trial populations by this attributable recurrence risk, which we hypothesize corresponds to stratification by expected-benefit. In performing Aim 5, it is important to recognize that subgroup analyses are prone to false positive results due to multiple comparisons. Indeed, an advantage of multivariable risk-based analysis is that it allows the influence of multiple variables to be summarized along a single dimension and thus minimize the risks of this Type I error. However, this is only the case if the stratification is *fully *specified, before the outcomes are examined, since even with a single risk model minor differences in stratification can influence results; when two risk models are combined, the potential for analytic flexibility-and therefore bias--is increased. While this Aim needs to be informed by the results of Aims 2 and 3, and the observed risk distributions in the RoPE populations, to emphasize the importance of *full pre*-specification of the stratification strategy, process and analysis before the outcome data of the randomized controlled trials (RCTs) are examined, we have partitioned this into a separate specific aim, to be completed before Aim 5.

A challenge of this step is that transformation of PFO propensity into a probability that a PFO is pathogenic versus incidental requires an estimate of the rate of PFO in a control population. The control rates of TEE-detected PFOs vary somewhat from study to study, presumably due to variation in TEE technique (since the presence of a PFO rates have been shown to be largely independent of patient characteristics in unselected [screened] populations[25,28]. Figure 1 shows the relationship of PFO propensity to the probability that a PFO is pathogenic under the assumptions that the control PFO rate is 15%, 20% and 25%. While the ranking of patients according to their probability that their PFO is pathogenically related to their index event is insensitive to the exact control rate selected, to converge on a control rate that would allow us to combine models #1 and #2, we will: 1) perform meta-analysis of control groups in prior case-control studies (CS versus non-cryptogenic stroke patients); 2) analyze the PFO-rate in TEE-studied non-cryptogenic stroke patients in component RoPE studies that included such patients (such as PICSS, which has 365 TEE-studied non-cryptogenic stroke patients). We will explore the distribution of the patient-specific probability that the discovered PFO is pathogenic across the different strata of the RoPE population.

Once the models are combined to produce the probability of stroke recurrence conditional on the probability that the index event was PFO-related (i.e. PFO-attributable recurrence risk), and based on a thorough understanding of the risk profile of the patients in the RoPE population and the randomized clinical trial population (but prior to any analysis of outcomes in the RCTs), the full stratification plan will be specified, as provisionally outlined in Aim 5.

### Aim 5: To apply this score to patients in completed clinical trials (currently on-going) testing percutaneous PFO closure against medical therapy, from low-expected-benefit to high-expected-benefit

The primary hypothesis of Aim 5 is that there will be a treatment-by-index interaction, when patients enrolled in RCTs testing PFO closure are ranked by their risk of stroke recurrence, conditioned on the probability that the PFO was pathogenically-related to the index event (using the composite index as a continuous variable). A p-value of 0.05 will be the threshold for significance. Depending on results, a clinically usable model will be developed. This step comprises external validation of the combined model. In addition, model 2 (the CS recurrence risk model) will be externally validated on the pooled medical arms of the RCTs by conventional criteria, including goodness-of-fit tests, ROC curve areas and calibration plots as described in the prior aims.

For Aim 5, we have, as of this writing, received agreement to test the risk models on two RCT databases, which will be pooled. Additional RCTs will be sought. The two RCTs to be pooled are:

Randomized Evaluation of Recurrent Stroke comparing PFO Closure to Established Current Standard of Care Treatment (RESPECT) Trial: (http://ClinicalTrials.gov identifier: NCT00465270) This is a prospective, randomized, multi-center trial, enrolling at 60 North American centers, designed to investigate whether percutaneous PFO closure with the Amplatzer PFO Occluder is superior to current standard of care medical treatment in the prevention of recurrent stroke. Inclusion criteria include age of 18-60 years old, cryptogenic stroke (not TIA) within 270 days, and TEE demonstration of a PFO. The recruitment target is 1000 subjects who will be randomized 1:1 to endovascular PFO closure or medical management. The primary endpoints are recurrence of non-fatal stroke, post-randomization death, and fatal ischemic stroke.

PC-Trial: Patent Foramen Ovale and Cryptogenic Embolism: (ClinicalTrials.gov identifier: NCT00166257). The PC-Trial is very similar in design to the RESPECT Trial comparing the efficacy of percutaneous PFO closure with medical treatment in 26 European centers. An additional inclusion criterion is cryptogenic peripheral embolism. Subjects are being stratified according to age and presence or absence of an atrial septal aneurysm. The recruitment target is 410 and the primary outcome is death, non-fatal stroke and peripheral embolism[29].

Adequacy of the Data for Aim 5: The proposed sample of 1400 subjects from RESPECT and the PC-Trial will provide sufficient power to detect a moderate interaction in the association of the recurrent risk stratification and the two study treatment groups. Power was estimated through simulations in SAS across multiple parameter configurations. For example, the proposed study sample of 700 subjects in each study arm, with an overall recurrence rate of 5% (stroke only), will provide approximately 80% power to detect a difference in the hazard ratio for a change of 10% in predicted risk of 1.00 and 1.33 for the two study groups, assuming a uniform distribution of risk scores. Additional RCTs will also be sought.

## List of Abbreviations

ASA: atrial septal aneurysm; CS: cryptogenic stroke; CT: computer tomography; ECG: electrocardiogram; MRI: magnetic resonance imaging; PE: paradoxical emboli; PFO: patent foramen ovale; RCT: randomized controlled trial; RESPECT Trial: Randomized Evaluation of Recurrent Stroke comparing PFO Closure to Established, Current Standard of Care Treatment Trial; ROC: receiver operator characteristics; RoPE: Risk of Paradoxical Embolism; TCD: transcranial Doppler; TEE: transesophageal echocardiography; TIA: transient ischemic attack; TOAST: Trial of ORG 10172 in Acute Stroke Treatment

## Competing interests

Drs. Kent and Thaler have no conflict of interests to report.

## Authors' contributions

Drs K and T co-developed the ideas for the RoPE Study and the RoPE protocol. Dr. K wrote the initial draft of this manuscript. Dr T revised the manuscript for important content. JG and RR provided statistical consultation. The RoPE Study Investigators (all authors) reviewed and approved the manuscript.
